# Development and applicability of Hospital Survey on Patient Safety Culture (HSOPS) in Japan

**DOI:** 10.1186/1472-6963-11-28

**Published:** 2011-02-07

**Authors:** Shinya Ito, Kanako Seto, Mika Kigawa, Shigeru Fujita, Toshihiko Hasegawa, Tomonori Hasegawa

**Affiliations:** 1 Department of Social Medicine, Toho University School of Medicine, Tokyo, Japan; 2 Department of Healthcare Management, Nippon Medical University, Tokyo, Japan

## Abstract

**Background:**

Patient safety culture at healthcare organizations plays an important role in guaranteeing, improving and promoting overall patient safety. Although several conceptual frameworks have been proposed in the past, no standard measurement tool has yet been developed for Japan.

**Methods:**

In order to examine possibilities to introduce the Hospital Survey on Patient Safety Culture (HSOPS) in Japan, the authors of this study translated the HSOPS into Japanese, and evaluated its factor structure, internal consistency, and construct validity. Healthcare workers (n = 6,395) from 13 acute care general hospitals in Japan participated in this survey.

**Results:**

Confirmatory factor analysis indicated that the Japanese HSOPS' 12-factor model was selected as the most pertinent, and showed a sufficiently high standard partial regression coefficient. The internal reliability of the subscale scores was 0.46-0.88. The construct validity of each safety culture sub-dimension was confirmed by polychoric correlation, and by an ordered probit analysis.

**Conclusions:**

The results of the present study indicate that the factor structures of the Japanese and the American HSOPS are almost identical, and that the Japanese HSOPS has acceptable levels of internal reliability and construct validity. This shows that the HSOPS can be introduced in Japan.

## Background

The past 10 years have witnessed an increasing interest in safety and quality issues in healthcare. Patient safety, including the measurement of patient safety culture, has become a top priority for health systems in developed nations [1].

Safety culture is defined as "the product of individual and group values, attitudes, perceptions, competencies, and patterns of behavior that determine the commitment to, and the style and proficiency of, an organization's health and safety management" [2]. Organizations with a favorable safety culture are characterized by communications founded on mutual trust, by shared perceptions of the importance of safety, and by confidence in the efficacy of preventive measures [2]. Implementation of a patient safety culture is thought to minimize adverse events and eliminate preventable harm in health care organizations.

A number of self-report questionnaires have been developed to measure the patient safety climate in hospitals [3-6]. One of these instruments is the Hospital Survey on Patient Safety Culture (HSOPS) developed by the US Agency for Healthcare Research and Quality (AHRQ) [7]. This questionnaire is composed of 42 items that are divided into subscales to measure 12 sub-dimensions of a safety culture. A 2004 US draft pilot survey of the HSOPS [7], examining 1,437 healthcare workers (female 81%; average age 43 years) of 21 hospitals (overall response rate 29%), reported that the tool showed acceptable levels of internal reliability (Cronbach's α = 0.63-0.84) and construct validity. Thereafter, the HSOPS became used nationwide in US health care facilities. In 2009, the AHRQ had 196,462 respondents from 622 participating hospitals in its comparative database. The characteristics of the 622 database hospitals are fairly consistent with the distribution of US hospitals registered with the American Hospital Association [7]. Most hospitals are nonteaching (69 percent) and non-government owned (voluntary/non-profit or proprietary/investor owned; 78 percent) [7]. The questionnaire has been translated into 16 different languages including Japanese, and it is currently used in 31 countries.

In terms of the factorial structure of the HSOPS, only two studies have replicated the factor structure of the HSOPS using the exploratory factor analysis (EFA). However, the results were different but only to some extent i.e. they found one less factor. For example, Blegen et al. [8] included 454 healthcare workers from three hospitals in their study, and reported that the tool was an 11-factor model, which did not include the staffing subscale, and showed to have moderate-to-strong reliability (Cronbach's α = 0.44-0.83) and validity. In Netherlands, Smits et al. [9] also reported an 11-factor model and moderate reliability (Cronbach's α = 0.57-0.79) and validity.

The purpose of this study is to examine the validity and applicability of the HSOPS in Japan and to compare the factor structure to that found in other studies, particularly the original US study. We translated the HSOPS into Japanese, and evaluated its internal consistency and construct validity.

## Methods

### Data Sources

This survey was conducted with healthcare workers from 13 acute care general hospitals in Japan over the period from January to November 2009. The participating hospitals included eight urban hospitals and five rural hospitals, one of which was a university hospital and the other 12 were teaching hospitals. Bed size numbers of these hospitals varied from 78 to 1,021 beds (three hospital <300, six hospital 300-500, and four hospital >500 beds). The Japanese version of the HSOPS (Japanese HSOPS) was distributed to all healthcare workers at each hospital through interoffice mail and returned by mail. To allow for confidentiality, all questionnaires were sealed in an envelope before collection. Additionally, the survey was conducted anonymously.

A total of 7,725 healthcare workers completed the questionnaire. A total of 9,867 questionnaires were originally sent out. Questionnaires (n = 629) in which participants selected "N/A" for an entire section were excluded from the analysis. A further 701 questionnaires in which fewer than half the items were answered were also excluded. The respondents' mean scores of all people on the item replaced missing values. The remaining 6,395 surveys were analysed. These surveys represented 74.9% of those distributed (54.6 to 92.9%; Table 1).

**Table 1:** Respondent and Hospital Characteristics

*Worker Characteristics*	*n (%)*		*Hospital Characteristics*	*n (%)*	
Job title			Bed size		
Nurse	3944	(61.7%)	Small (0-299 beds)	3	(23.1%)
Administrative worker	682	(10.7%)	Med (300-500 beds)	6	(46.2%)
Physician	538	(8.4%)	Large (500 > beds)	4	(30.8%)
Technician	481	(7.5%)	Teaching status		
Dietician	202	(3.2%)	Teaching hospital	12	(92.3%)
Pharmacist	155	(2.4%)	University hospital	1	(7.7%)
Therapist*	116	(1.8%)	Location		
Janitor	32	(0.5%)	Urban	8	(61.5%)
Other	208	(3.3%)	Rural	5	(38.5%)
No answer	37	(0.6%)			
Age					
<20	25	(0.4%)			
20-29	2187	(34.2%)			
30-39	1761	(27.5%)			
40-49	1107	(17.3%)			
50-59	772	(12.1%)			
>59	346	(5.4%)			
No answer	197	(3.1%)			
Gender					
Female	4830	(75.5%)			
Male	1450	(22.7%)			
No answer	115	(1.8%)			

*Therapist is Physical/Occupational/Speech/Orthoptics therapist.

### Hospital Survey on Patient Safety Culture

First, permission was obtained from the author to use the HSOPS. Then, the HSOPS was translated to Japanese by a panel including a bilingual English-Japanese translator and specialists in patient safety. Although no back-translation was conducted, the Japanese HSOPS was given to other experts in safety culture, who verified the accuracy of the translation.

The Japanese HSOPS uses Likert scales with six response options ranging from "Strongly Disagree", "Disagree", "Neither", "Agree", "Strongly Agree", and "N/A". The original US HSOPS does not offer the option "N/A", but several of its questions do not cover the Japanese situation, and the specialist panel decided to add the "N/A" option. The Japanese HSOPS consists of the same 12 sub-domains as the study by the AHRQ [7]: (1) Frequency of Event Reporting, (2) Overall Perceptions of Safety, (3) Supervisor/Manager Expectations & Actions Promoting Safety, (4) Organizational Learning-Continuous Improvement, (5) Teamwork within Hospital Units, (6) Communication Openness, (7) Feedback and Communication about Error, (8) Nonpunitive Response to Error, (9) Staffing, (10) Hospital Management Support for Patient Safety, (11) Teamwork Across Hospital Units, and (12) Hospital Handoffs & Transitions. Additionally two further single-item measures of outcomes are included in the HSOPS; the respondents' ratings of patient safety in their workplaces (Patient Safety Grade; Please give your work area/unit in this hospital an overall grade on patient safety) and the number of adverse events they had reported in the last 12 months (Number of Events Reported; in the past 12 months, how many event reports have you filled out and submitted?).

### Data Analyses

A three-step analysis was conducted. For the first step, to assess the suitability of the Japanese data for the preceding studies [7-9] and examine the dimensionality of the survey, a series of confirmatory factor analyses (CFA) were carried out. The estimation method was the maximum likelihood procedure. Nine statistics were used to assess the best model fit [10,11]: the chi-square, the comparative fit index (CFI), the Tucker-Lewis index (TLI), the root mean square error of approximation (RMSEA), its 90% confidence interval (CI), and the standardized root mean square (SRMR), the Akaike information criterion (AIC), the consistent Akaike information criterion (CAIC), and the Bayesian information criterion (BIC).

The chi-square test assesses the magnitude of the discrepancy between the sample and the fitted covariance matrix. A non-statistically significant chi-square value indicates a good model fit. Although the chi-square is very sensitive to sample size, the CFI provides a measure of proportional increase in fit over a null model. CFI varies from 0 to 1 and a CFI value of >0.90 indicates a good model fit. The TLI, also known as Non-Normed Fit Index (NNFI), combines a measure of parsimony into a comparative index between the proposed and null models, resulting in values ranging from 0 to 1. RMSEA values of 0.05 or less indicate a reasonable error of approximation in a population. The SRMR is the average discrepancy between the correlations observed in the input matrix and those predicted by the model. SRMR can take a range of values between 0 and 1, with 0 indicating a perfect fit. The AIC permits the comparison of non-nested models. Generally, models with the lowest AIC are judged to fit the data better than alternative solutions. The CAIC measures the global fit of a cluster model to an input data, and the smallest CAIC value suggests the best fit. The Bayesian information criterion (BIC) or Schwarz Criterion (also SBC, SBIC) is a criterion for model selection among a class of parametric models with different numbers of parameters. The model with the lowest BIC is preferred. Secondly, Cronbach's coefficient α was calculated to measure internal consistency.

For the third step, to verify construct validity, polychoric correlations were calculated for the 12 safety culture sub-dimensions which are the ordinal variables. Polychoric correlation is analogue to Pearson's correlation analysis; however, polychoric correlation is the correlation between ordinal variables, whereas Pearson's correlation is the correlation between continuous variables. In addition, to verify construct validity, an ordered probit analysis was conducted between the 2 single-item measures of outcome (Patient Safety Grade and Number of Events Reported, which are the ordinal variables) and the 12 safety culture sub-dimensions. In each analysis, Patient Safety Grade and Number of Events Reported were the dependent variables; the 12 safety culture sub-dimensions were the independent variables. Ordered probit analysis is analogue to multiple regression analysis; however, in the ordered probit the dependent variable is scored on an ordinal scale, whereas in a multiple regression the dependent variable is scored on an interval scale. The use of multiple regression analysis with an ordinal dependent variable results in biased estimates of the parameters and standard errors. Furthermore, polychoric correlations were calculated between 2 single-item measures of outcome and the 12 safety culture sub-dimensions. The Mplus (Mplus version 3.0) was used for confirmatory factor analysis, polychoric correlation and ordered probit analysis. Descriptive statistics and internal consistency reliability coefficients were performed with the use of the open-source R software, version 2.8.1.

## Results

### Confirmatory Factor Analysis and Internal Reliability

For the Japanese HSOPS, a 12-factor model was selected as the most pertinent (χ^2 ^= 11035, *df *= 753, CFI = 0.89, TLI = 0.88, RMSEA = 0.046, RMSEA 90% CI = 0.045-0.047, SRMR = 0.044, AIC = 9529.5, CAIC = 3683.7, BIC = 4436.7), showing a sufficiently high standard partial regression coefficient. Although Blegen et al.'s [8] 11 factor model showed acceptable measurement properties (χ^2 ^= 15827, *df *= 648, CFI = 0.83, TLI = 0.82, RMSEA = 0.061, RMSEA 90% CI = 0.060-0.061, SRMR = 0.150, AIC = 14531.4, CAIC = 9500.8, BIC = 10148.8), Smits et al.'s [9] 11 factor model did not (χ^2 ^= 59819, *df *= 806, CFI = 0.38, TLI = 0.34, RMSEA = 0.107, RMSEA 90% CI = 0.106-0.108, SRMR = 0.392, AIC = 58207.0, CAIC = 51949.8, BIC = 52755.8). The α coefficient for the Japanese HSOPS of the total score was 0.92. Cronbach's α values for the subscale scores were 0.46-0.88 (Table 2).

**Table 2:** Mean Factor Scores, Cronbach α Coefficient, and Polychoric Correlations of the Japanese HSOPS

		*M*	*SD*	*α*	*F1*	*F2*	*F3*	*F4*	*F5*	*F6*	*F7*	*F8*	*F9*	*F10*	*F11*
F1.	Frequency of Event Reporting	11.9	2.97	.88	-										
F2.	Overall Perceptions of Safety	13.8	2.48	.62	.20	-									
F3.	Supervisor/Manager Expectations & Actions Promoting Safety	14.7	2.55	.70	.24	.45	-								
F4.	Organizational Learning Continuous Improvement	10.4	1.84	.65	.22	.51	.44	-							
F5.	Teamwork within Hospital Units	14.9	2.66	.83	.20	.45	.45	.51	-						
F6.	Communication Openness	10.4	2.19	.62	.29	.46	.49	.47	.52	-					
F7.	Feedback and Communication about Error	10.7	2.31	.77	.37	.45	.46	.56	.44	.60	-				
F8.	Nonpunitive Response to Error	9.6	2.32	.71	.14	.39	.39	.25	.37	.41	.27	-			
F9.	Staffing	12.1	2.59	.46	.04	.39	.25	.11	.22	.20	.14	.36	-		
F10.	Hospital Management Support for Patient Safety	10.3	1.96	.61	.22	.54	.49	.49	.41	.45	.46	.33	.29	-	
F11.	Teamwork Across Hospital Units	13.1	2.46	.70	.15	.48	.39	.42	.44	.43	.38	.34	.26	.57	-
F12.	Hospital Handoffs & Transitions	12.7	2.43	.73	.09	.42	.29	.28	.28	.32	.28	.30	.29	.43	.57

### Construct Validity

Polychoric correlations were calculated for the 12 safety culture sub-dimensions (Table 2). Positive correlation was found for each pair of subgroup scores, although several correlations were small. The highest correlation (*r *= 0.60) was shown between Communication Openness and Feedback and Communication about Error. The lowest correlation (*r *= 0.04) was shown between Frequency of Event Reporting and Staffing. Regarding the relationships between outcome variables and safety culture sub-dimensions, Frequency of Event Reporting showed small correlations to other safety culture sub-dimensions (*r *= 0.04-0.37). Overall, Perceptions of Safety showed medium correlations (*r *= 0.39-0.54).

To verify construct validity, ordered probit analysis was conducted (Table 3). In regard to Patient Safety Grade, middle standard partial regression coefficients were found towards all 12 safety culture sub-dimensions. Overall Perceptions of Safety had a moderate standard partial regression coefficient (β = 0.28). The R square value was 0.44. In regard to Number of Events Reported, small standard partial regression coefficients were generally found. The R square value was 0.06. Thereafter, polychoric correlations were calculated (Table 4). The Patient Safety Grade showed middle or large correlations compared to the 12 safety culture sub-dimensions (*r *= 0.23-0.58). In regard to Number of Events Reported, all correlations were very small (*r *= -0.12-0.17).

**Table 3:** Mean Scores and Standard Partial Regression Coefficient of the Items of the Japanese HSOPS

		*M*	*SD*	*F1*	*F2*	*F3*	*F4*	*F5*	*F6*	*F7*	*F8*	*F9*	*F10*	*F11*	*F12*
D1	When a mistake is made, but is caught and corrected before affecting the patient, how often is this reported?	3.71	1.1	.70											
D2	When a mistake is made, but has no potential to harm the patient, how often is this reported?	4.05	1.1	.99											
D3	When a mistake is made that could harm the patient, but does not, how often is this reported?	4.13	1.1	.86											
A15	Patient safety is never sacrificed to get more work done.	3.42	0.9		.44										
A18	Our procedures and systems are good at preventing errors from happening.	3.49	0.8		.58										
A10*	It is just by chance that more serious mistakes don't happen around here.	3.36	1.0		.55										
A17*	We have patient safety problems in this unit.	3.54	0.9		.61										
B1	My supervisor/manager says a good word when he/she sees a job done according to established patient safety procedures.	3.12	0.9			.55									
B2	My supervisor/manager seriously considers staff suggestions for improving patient safety.	3.45	0.9			.75									
B3*	Whenever pressure builds up, my supervisor/manager wants us to work faster, even if it means taking shortcuts.	4.00	0.9			.49									
B4*	My supervisor/manager overlooks patient safety problems that happen over and over.	4.10	0.9			.65									
A6	We are actively doing things to improve patient safety.	3.75	0.8				.66								
A9	Mistakes have led to positive changes here.	3.52	0.8				.64								
A13	After we make changes to improve patient safety, we evaluate their effectiveness.	3.17	0.8				.55								
A1	People support one another in this unit.	3.90	0.8					.82							
A3	When a lot of work needs to be done quickly, we work together as a team to get the work done.	3.77	0.8					.81							
A4	In this unit, people treat each other with respect.	3.49	0.9					.71							
A11	When one area in this unit gets really busy, others help out.	3.74	0.9					.64							
C2	Staff will freely speak up if they see something that may negatively affect patient care.	3.42	0.9						.62						
C4	Staff feel free to question the decisions or actions of those with more authority.	3.38	1.0						.65						
C6*	Staff are afraid to ask questions when something does not seem right.	3.57	1.0						.52						
C1	We are given feedback about changes put into place based on event reports.	3.28	0.9							.63					
C3	We are informed about errors that happen in this unit.	3.86	0.9							.77					
C5	In this unit, we discuss ways to prevent errors from happening again.	3.58	0.9							.80					
A8*	Staff feel like their mistakes are held against them.	2.90	1.0								.68				
A12*	When an event is reported, it feels like the person is being written up, not the problem.	3.13	1.0								.78				
A16*	Staff worry that mistakes they make are kept in their personnel file.	3.58	0.9								.56				
A2	We have enough staff to handle the workload.	2.58	1.1									.44			
A5*	Staff in this unit work longer hours than is best for patient care.	2.71	1.1									.36			
A7*	We use more agency/temporary staff than is best for patient care.	3.76	1.0									.19			
A14*	We work in "crisis mode," trying to do too much, too quickly.	3.08	1.0									.79			
F1	Hospital management provides a work climate that promotes patient safety.	3.63	0.8										.63		
F8	The actions of hospital management show that patient safety is a top priority.	3.39	0.9										.61		
F9*	Hospital management seems interested in patient safety only after an adverse event happens.	3.23	1.0										.53		
F4	There is good cooperation among hospital units that need to work together.	3.47	0.8											.61	
F10	Hospital units work well together to provide the best care for patients.	3.47	0.8											.67	
F2*	Hospital units do not coordinate well with each other.	2.96	0.9											.61	
F6*	It is often unpleasant to work with staff from other hospital units.	3.24	0.9											.56	
F3*	Things "fall between the cracks" when transferring patients from one unit to another.	3.03	0.9												.63
F5*	Important patient care information is often lost during shift changes.	3.23	0.8												.66
F7*	Problems often occur in the exchange of information across hospital units.	3.15	0.8												.72
F11*	Shift changes are problematic for patients in this hospital.	3.25	0.8												.53

Note: A1 through A18, B1 through B4, C1 through C6, D1 through D3, and F1 through F22 indicate the question number of HSOPS.

*This is the reverse worded. The means reported have been reverse scored.

**Table 4:** Polychoric Correlations and Standard Partial Regression Coefficient of the Japanese HSOP

	*Patient Safety Grade*		*Number of Events Reported*	
		
	r*	**β**^**†**^	r*	**β**^**†**^
Frequency of Event Reporting	.23	.06	.17	.14
Overall Perceptions of Safety	.58	.28	-.06	-.08
Supervisor/Manager Expectations & Actions Promoting Safety	.42	.05	.03	.03
Organizational Learning Continuous Improvement	.49	.15	.05	.04
Teamwork Within Hospital Units	.41	.05	.00	-.01
Communication Openness	.42	.03	.01	-.07
Feedback and Communication About Error	.44	.05	.12	.15
Nonpunitive Response To Error	.29	-.03	-.03	.01
Staffing	.29	.07	-.12	-.08
Hospital Management Support for Patient Safety	.51	.13	-.02	-.02
Teamwork Across Hospital Units	.44	.04	-.04	-.01
Hospital Handoffs & Transitions	.38	.07	-.08	-.05

*r represents polychoric correlation

^†^β represents standard partial regression coefficient

## Discussion

This study produced three major findings. First, the AHRQ's 12 factor model provides the best fit to the Japanese HSOPS data for acute care hospital staffs compared with the two 11 factor models proposed in the previous studies [8,9]. Second, the Japanese HSOPS have shown acceptable internal consistency for the subscales, although the Staffing factor has shown low internal consistency. Finally, the construct validity of each safety culture sub-dimensions is confirmed by the polychoric correlations between the 12 safety culture sub-dimensions, and by the ordered probit analysis for the 2 outcomes and 12 safety culture sub-dimensions. The results indicate that the HSOPS can be introduced in Japan.

In terms of the confirmatory factor analysis, the structure underlying the hospital safety culture in Japan fits the same 12 factor model as the study by AHRQ [7]. In addition, the 12 factor model has shown a sufficiently high standard partial regression coefficient. These results again suggest that the Japanese HSOPS should include 12 sub-dimensions and 42 items. However these results are not in line with those reported by Blegen et al. [8] and Smiths et al. [9] that the tool was an 11-factor model. The Japanese HSOPS has shown acceptable internal consistency for the subscales. However, they generally show a lower internal consistency than in the AHRQ study; the Staffing scale being particularly low. These results are congruent with those reported by the preceding studies [8,9].

The reasons of the different results of previous and this study as well as of the low internal consistency are unclear but may be related to the fact that there is a limited number of question items which constitute the each factor. This result suggests that it is necessary to investigate new question items to compliment factors with low internal consistency.

The Japanese HSOPS has also shown acceptable construct validity. First, the moderate positive correlations between the 12 factors suggest that each of the safety culture sub-dimensions measures a similar construct, although Frequency of Event Reporting, one of the outcome variables, has actually only a small relationship with the other safety culture sub-dimensions. Secondly, the ordered probit analysis shows that the 12 safety culture sub-dimensions can estimate the Patient Safety Grade, one of the single-item measures of outcomes.

These findings point to the important role that the Japanese HSOPS could play in the assessment of patient safety culture. On the other hand, the 12 safety culture sub-dimensions did not show a relationship between the Number of Events Reported. Our results concur with AHRQ's report that the relationships between the Number of Events Reported and the safety culture dimensions were very small [7]. One explanation for the lack of relationship with Number of Events Reported is that the proportion of respondents with reporting experience was very low; approximately 72% of the respondents reported "no events" or "1 to 2 events" in the last 12 months.

The findings of this study should be considered in light of its limitations.

First, the research was conducted on 6,395 questionnaire replies from 13 general hospitals. Although the present study was more representative of the population of hospital workers than any prior studies on patient safety culture, this number may limit a generalization of its findings to other general hospitals in Japan. In particular, the occupation groups were disproportionate number of participants. Future research should seek to conduct random samplings.

Second, although our response rate was comparatively high (74.9%), the number of eliminated data was also large, mainly because a high rate of respondents selected "N/A" to questions (6.4%). This suggests that some of the survey's items are not suitable for all acute care hospitals.

Third, the present study did not evaluate relationships with objective indicators such as a rate of incidents and a number of reports by some type of error. Further studies are needed to compare the Japanese HSOPS with these objective indicators.

Fourth, the present study used an analytical approach suitable for ordinal scales. Pearson's correlation and multiple regression analysis with ordinal variables, which were used in previous studies, are known to result in biased estimates of the parameters. The present study therefore used polychoric correlation and ordered probit analysis. The different approaches may make it difficult to compare results directly.

Finally, this study did not evaluate the test-retest reliabilities of the Japanese HSOPS. In order to confirm any change of the Japanese HSOPS over time reflecting the situation of the hospital, future studies need to use a longitudinal design.

## Conclusions

The results of the present study support the view that the Japanese HSOPS has a valuable role in the assessment of the patient safety culture in Japan. The factor structures of the Japanese and US HSOPS are almost identical, and perform better than the 11 factor models found by preceding studies [8,9]. The Japanese factors show acceptable internal consistency for the subscales, although the internal consistency of this study is low compared to that of the US study. Particularly, the Staffing factor has shown only small internal consistency. The reason for this is unclear but may be related to the fact that there is a limited number of question items which constitute each factor. The Japanese HSOPS has shown acceptable construct validity. The safety culture sub-dimensions measure a similar construct, and show the relationship between Patient Safety Grade and one of the outcomes. However, not all safety culture sub-dimensions show a relationship with Number of Events Reported. This result was the same as with the previous study by the AHRQ [7]. To further investigate these differences, future studies using random sampling and cross-national design are needed.

## Competing interests

The authors declare that they have no competing interests.

## Authors' contributions

SI performed the statistical analysis and prepared the draft. KS, MK and SF acquired the data, participated in the design of the study and performed the statistical analysis. TH and TH managed the whole research processes. All authors read and approved the final manuscript.

## Pre-publication history

The pre-publication history for this paper can be accessed here:

http://www.biomedcentral.com/1472-6963/11/28/prepub
